# Socio-economic impact of antimicrobial resistance in Pakistan: a comprehensive review

**DOI:** 10.3389/fpubh.2026.1809327

**Published:** 2026-06-09

**Authors:** Zernila Zaheer

**Affiliations:** 1 Faculty of Life Sciences, Hamburg University of Applied Sciences, Hamburg, Germany; 2 Department of Food, Nutrition and Health, University of Bayreuth, Bayreuth, Germany

**Keywords:** antimicrobial resistance, antimicrobial stewardship, healthcare costs, national action plan, Pakistan, public health policy, socioeconomic burden, surveillance

## Abstract

Antimicrobial resistance (AMR) is an increasing public health and economic threat, with substantial consequences for low- and middle-income countries such as Pakistan. This review critically synthesizes peer-reviewed evidence on the socioeconomic impact of AMR in Pakistan and examines policy and implementation challenges linked to the National Action Plan on AMR. A structured PubMed search was conducted for studies published between January 2025 and April 2026, with the final search completed on 28 April 2026. Search terms included “antimicrobial resistance AND Pakistan,” “AMR AND Pakistan AND National Action Plan,” “antimicrobial resistance AND socioeconomic impact AND Pakistan,” “antibiotic resistance AND healthcare costs AND Pakistan,” and “AMR AND surveillance AND Pakistan.” Following PRISMA-guided screening, 29 studies were included in the qualitative synthesis. The findings indicate rising resistance across multiple pathogens and settings, including enteric pathogens, respiratory organisms, multidrug-resistant Gram-negative bacteria, and resistant infections in hospital, community, and environmental contexts. Evidence from genomic and epidemiological studies shows persistent fluoroquinolone resistance and emerging azithromycin resistance, suggesting narrowing therapeutic options. The socioeconomic burden includes prolonged illness, increased diagnostic and treatment costs, longer hospital stays, productivity loss, and household financial strain. However, national cost-of-illness evidence remains limited, and available estimates are mainly derived from single-center studies. Policy gaps include fragmented surveillance, weak enforcement of prescription regulations, inconsistent antimicrobial stewardship, and limited public awareness. Strengthening integrated surveillance, improving regulatory enforcement, expanding vaccination strategies, and embedding AMR within health financing and public health planning are essential to reduce the long-term socioeconomic burden of AMR in Pakistan.

## 1 Introduction

Antimicrobial resistance (AMR) is a growing global health threat, undermining the efficacy of essential treatments and increasing both morbidity and mortality. Low- and middle-income countries, including Pakistan, are disproportionately affected due to limited healthcare resources, high infectious disease burden, and widespread antibiotic misuse (33).

In Pakistan, AMR is observed across multiple pathogens, including *Salmonella enterica, Vibrio cholerae, Klebsiella pneumoniae*, and multidrug-resistant Gram-negative and Gram-positive bacteria in both community and healthcare settings. The economic burden is significant, encompassing direct medical costs, prolonged hospital stays, and indirect costs such as productivity loss. The National Action Plan (NAP) for AMR in Pakistan, implemented over the last 5 years, has faced challenges in surveillance, enforcement, and public awareness, highlighting the need for evidence-based interventions.

This review synthesizes the current literature on AMR in Pakistan, examining prevalence, trends, and socioeconomic impacts to guide policy, stewardship, and public health initiatives.

## 2 Methods

A structured literature review was conducted using PubMed as the primary database to identify peer-reviewed studies related to antimicrobial resistance (AMR) in Pakistan. The search covered publications from January 2025 to April 2026, and the final search was conducted on 28 April 2026.

### 2.1 Search strategy

The following search terms and Boolean operators were used:

- “Antimicrobial resistance” AND “Pakistan”
- “AMR” AND “Pakistan” AND “National Action Plan”
- “Antimicrobial resistance” AND “socioeconomic impact” AND “Pakistan”
- “Antibiotic resistance” AND “healthcare costs” AND “Pakistan”
- “‘AMR” AND “surveillance” AND “Pakistan”

The search term “Antimicrobial resistance AND Pakistan” generated approximately 3,550 results, while “AMR AND Pakistan AND National Action Plan” generated approximately 1,140 results. Additional records were identified through manual screening of reference lists from eligible articles.

Eligibility criteria:

Studies were included if they:

- focused on human health;
- reported Pakistan-specific AMR findings;
- addressed socioeconomic burden, healthcare system impacts, antimicrobial stewardship, surveillance, or policy implementation;
- were peer-reviewed full-text journal articles published in English.

Animal and environmental studies were included only when they demonstrated direct One Health relevance to human antimicrobial resistance transmission, surveillance, or public health implications in Pakistan.

Studies were excluded if they:

- focused exclusively on animals or veterinary AMR without human public health relevance;
- were conducted outside Pakistan without contextual relevance;
- were conference abstracts, editorials, theses, posters, or unpublished reports;
- lacked full text or peer review.

Studies were excluded if they focused exclusively on animals or veterinary antimicrobial resistance without direct relevance to human health. However, animal, food-chain, and environmental studies were included when they demonstrated direct One Health relevance to antimicrobial resistance transmission, surveillance, environmental dissemination, or public health implications in Pakistan.

The inclusion of selected environmental and food-chain studies was guided by the One Health framework, recognizing that resistant organisms and resistance genes may circulate between humans, animals, food systems, and environmental reservoirs.

### 2.2 Study screening and selection

Titles and abstracts were screened manually for relevance. Duplicate articles were removed prior to full-text assessment. The majority of excluded studies were removed because they focused exclusively on veterinary AMR, lacked Pakistan-specific data, were non-peer-reviewed, or did not address socioeconomic or public health implications.

The review process followed Preferred Reporting Items for Systematic Reviews and Meta-Analyses (PRISMA) guidelines. The PRISMA study selection process is summarized in Table 1.

**Table 1:** PRISMA flow diagram illustrating the identification, screening, eligibility assessment, and inclusion process of studies related to antimicrobial resistance and its socioeconomic impact in Pakistan.

PRISMA stage	Description	Number of records
Identification	Records identified through PubMed database searching	4,714
Identification	Additional records identified through manual reference screening	29
Identification	Total records identified	4,743
Screening	Records after duplicate removal	4,000
Screening	Records screened by title and abstract	4,000
Screening	Records excluded during title and abstract screening	3,900
Eligibility	Full-text articles assessed for eligibility	100
Eligibility	Full-text articles excluded	71
Eligibility	Reasons for exclusion	Non-human studies; non-Pakistan studies; animal-only AMR studies; conference abstracts; editorials; non-peer-reviewed articles; studies without socioeconomic or public health relevance
Included	Studies included in qualitative synthesis	29

#### 2.2.1 . Data extraction and content analysis

Data extraction focused on:

- pathogen type;
- resistance mechanisms;
- geographic distribution;
- healthcare system impacts;
- direct and indirect economic burden;
- antimicrobial stewardship challenges;
- infection prevention and control measures;
- surveillance limitations;
- policy implementation barriers.

A qualitative content analysis approach was used to synthesize findings across studies. Thematic categories included:

- direct healthcare costs;
- indirect productivity losses;
- surveillance and stewardship gaps;
- environmental and One Health transmission pathways;
- healthcare infrastructure limitations;
- policy implementation challenges.

Inclusion criteria were:

(1) studies conducted in Pakistan,
(2) human clinical or public health research,
(3) peer-reviewed journal publications,
(4) English language articles.

Exclusion criteria included studies conducted outside Pakistan, research focusing exclusively on animals, birds, or non-human subjects, unpublished manuscripts, conference proceedings, abstracts without full text, theses, seminar reports, and non-English publications.

Data extraction focused on study design, geographic location within Pakistan, reported resistance patterns, and socioeconomic implications related to healthcare costs, productivity loss, treatment complexity, or health system strain.

## 3 Results

### 3.1 Comparative synthesis of included studies

**Figure 1:**
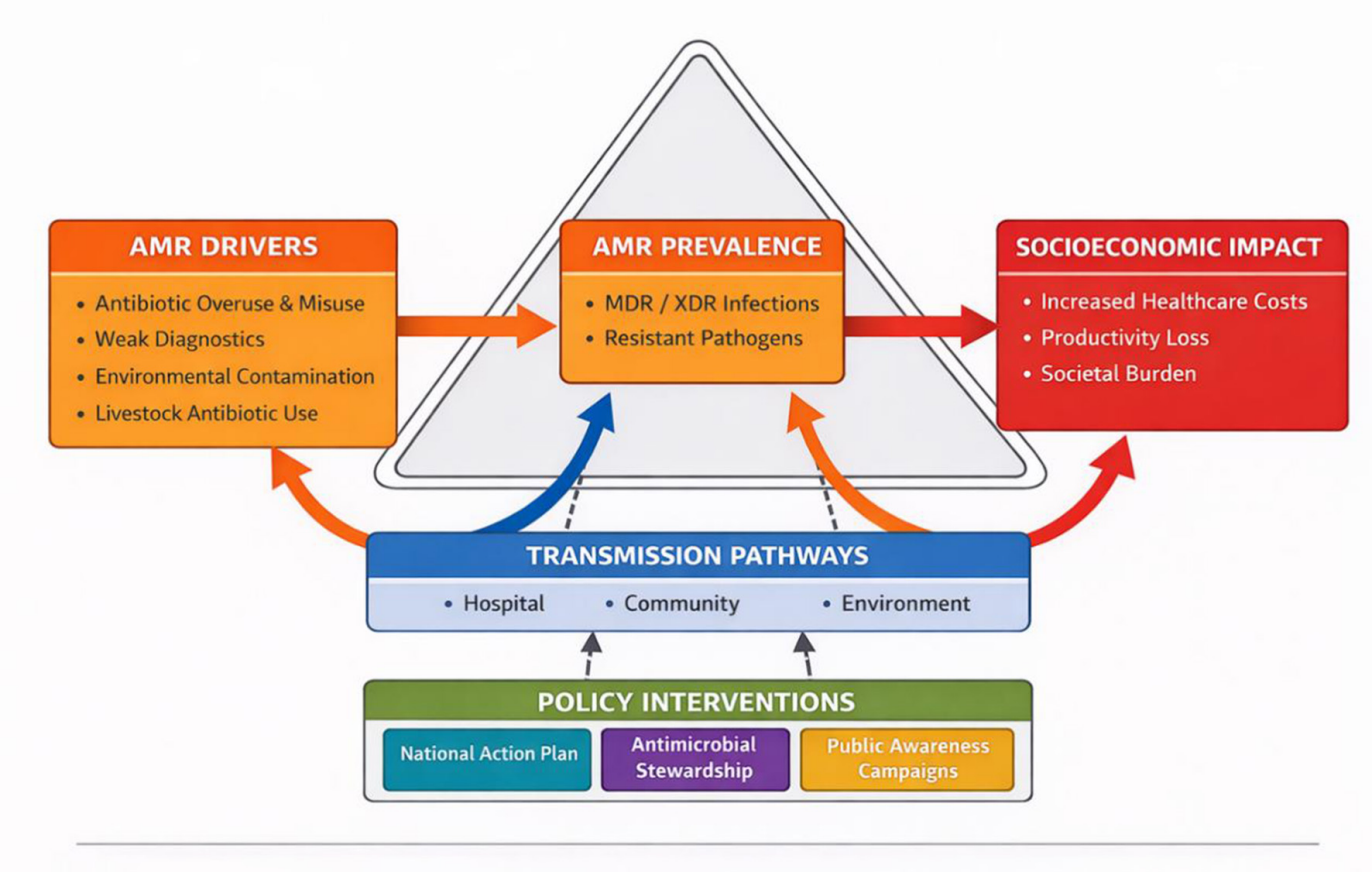
Conceptual framework illustrating drivers, transmission pathways, healthcare consequences, and socioeconomic impacts of antimicrobial resistance in Pakistan. This figure was developed by the author based on synthesized evidence from included studies.

**Figure 2:**
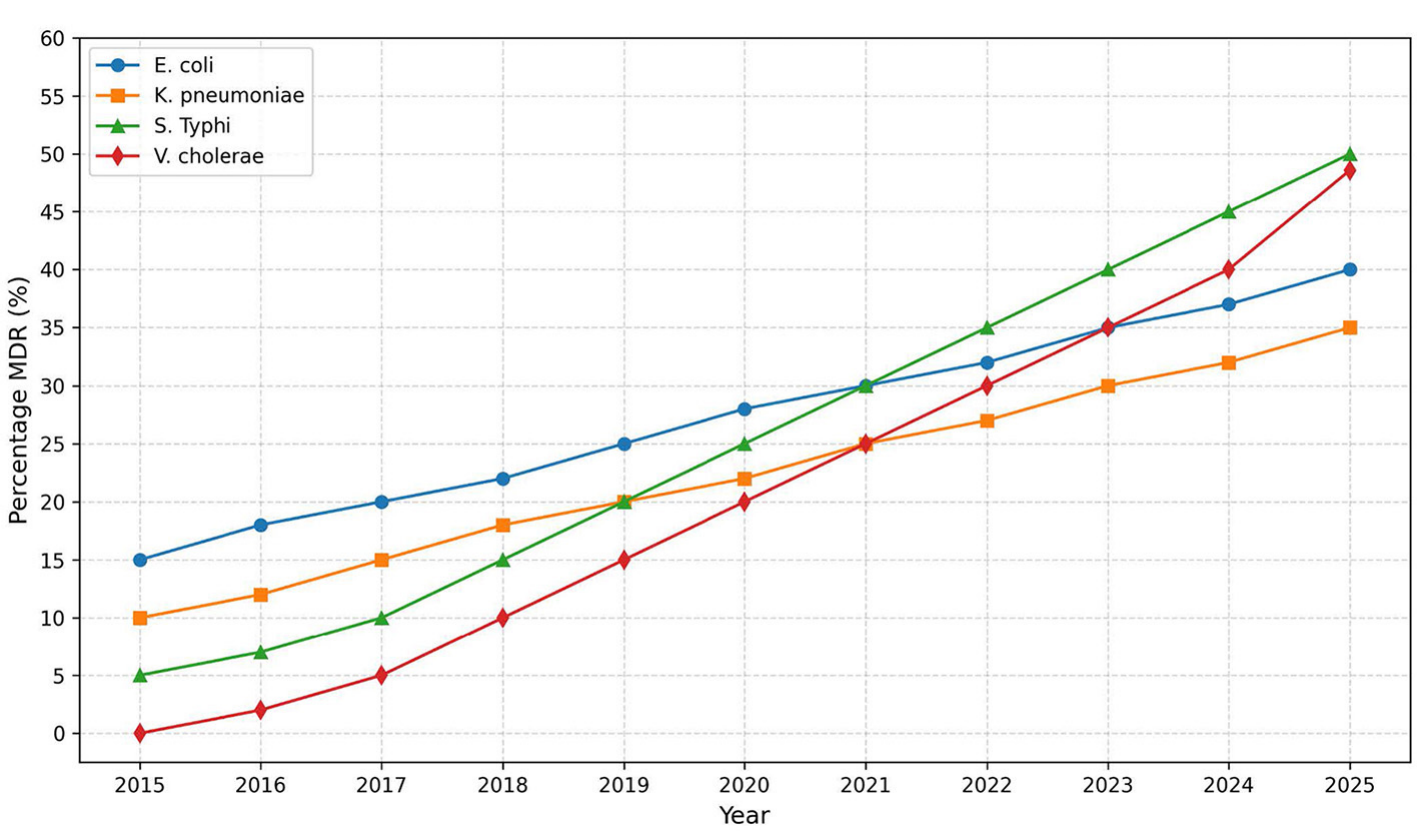
Epidemiological trends in multidrug resistance among major bacterial pathogens reported in Pakistan between 2015 and 2025. The figure represents a qualitative synthesis of published surveillance and clinical studies included in this review and does not represent pooled national surveillance estimates.

**Figure 3:**
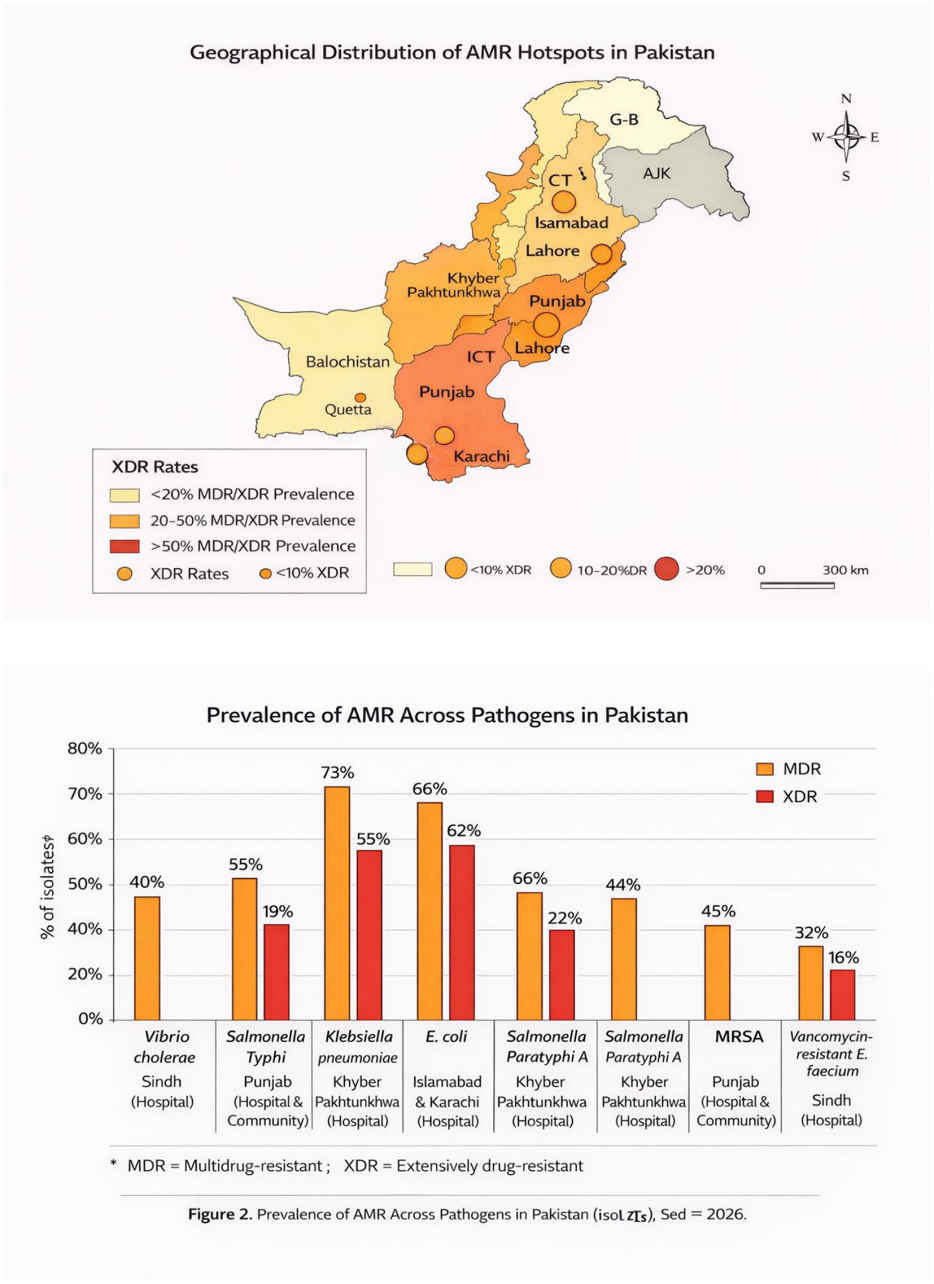
Prevalence of AMR across pathogens in Pakistan (isol ZIs), Sed = 2026.

The conceptual relationship between AMR drivers, transmission pathways, policy interventions, and socioeconomic impact is illustrated in Figure 1, while Figure 2 summarizes reported multidrug resistance trends among major pathogens. Figure 3 presents the geographical distribution and pathogen-specific prevalence of AMR across Pakistan. Although the included studies consistently report increasing antimicrobial resistance across Pakistan, important differences exist between hospital-based, community-based, and environmental surveillance studies. Hospital-based studies, particularly those conducted in tertiary-care and intensive-care settings, reported substantially higher multidrug resistance rates due to repeated antibiotic exposure and critically ill patient populations (32, 35).

In contrast, environmental and One Health studies primarily identified contamination pathways associated with wastewater exposure, livestock practices, and inappropriate antimicrobial use outside healthcare facilities. Despite methodological heterogeneity, most studies agreed that inadequate antimicrobial stewardship, weak surveillance systems, and irrational antibiotic use remain major contributors to rising AMR trends in Pakistan.

Recent genomic and epidemiological studies reveal a consistent pattern of rising antimicrobial resistance across multiple pathogens in Pakistan. The genomic analysis of 354 Salmonella Paratyphi A isolates collected from three provinces between 2017 and 2022 demonstrated dominance of specific genotypes and widespread fluoroquinolone-associated gyrA mutations (1). Although multidrug resistance was not widespread in this cohort, the detection of azithromycin resistance–associated mutations indicates potential narrowing of therapeutic options.

Similarly, respiratory infection surveillance data indicate growing resistance trends in clinically significant bacterial pathogens (2). Other recent microbiological and genomic investigations conducted in different provinces of Pakistan report resistance to commonly prescribed antibiotics, suggesting geographic heterogeneity but national relevance.

From a socioeconomic perspective, these resistance patterns translate into prolonged hospital stays, increased diagnostic requirements, escalation to second- or third-line antimicrobials, and higher direct medical costs. Indirect costs include lost wages, caregiver burden, and reduced productivity. In settings where out-of-pocket expenditure remains high, these financial pressures disproportionately affect lower-income households.

Despite the establishment of Pakistan's National Action Plan on AMR, implementation challenges persist. Surveillance systems remain fragmented, antimicrobial stewardship programs are inconsistently enforced, and regulatory control over antibiotic sales remains limited.

### 3.2 Major resistant pathogens in Pakistan

Extensively drug-resistant (XDR) *Salmonella Typhi* has emerged as a major antimicrobial resistance concern in Pakistan. A retrospective study from Karachi reported increasing resistance to first-line antibiotics and third-generation cephalosporins, substantially limiting treatment options and increasing dependence on azithromycin and carbapenems (3). The emergence of XDR typhoid reflects broader antimicrobial misuse, inadequate sanitation infrastructure, and surveillance gaps contributing to the growing public health burden in Pakistan (3). The characteristics and findings of the included studies are summarized in Table 2.

**Table 2:** Synthesis of included studies.

References	Article title	Study design	Study setting (Pakistan)	Main findings
Ali et al. (4)	Whole genome sequencing of drug-resistant vibrio cholerae serotype Ogawa from an outbreak in Khyber Pakhtunkhwa.	Laboratory-based cross-sectional outbreak investigation with WGS.	Khyber Pakhtunkhwa Province (Dir and Peshawar)	70 V. cholerae Ogawa identified; high AMR rates to ampicillin, sulfamethoxazole-trimethoprim, erythromycin, tetracycline; 48.6% MDR, 18.6% XDR; genomic determinants detected; persistent and internationally related lineages.
Abdullah et al. (13)	XDR typhoid in Pakistan: a threat to global health security and a wake-up call for antimicrobial stewardship.	Commentary/evidence-based perspective.	Sindh Province (Karachi) and national context	XDR *S. Typhi* is a major AMR threat; linked to antibiotic misuse, weak diagnostics, poor WASH; risk of international dissemination; calls for stewardship and improved infrastructure.
Safdar et al. (9)	Economic burden of antimicrobial resistance on patients in Pakistan.	Prospective cohort study	Islamabad & Rawalpindi (ICT & Punjab)	AMR bloodstream infections incurred ~USD 33.97 higher costs than susceptible cases; indirect costs (productivity loss, food, accommodation) were significant.
Mylona et al. (1)	A contemporary genomic snapshot of Salmonella Paratyphi A in Pakistan.	Genomic surveillance and phylogenomic analysis	Three provinces in Pakistan (2017–early 2022)	Analysis of 354 isolates showed pervasive fluoroquinolone-associated mutations; no MDR detected; one isolate with azithromycin resistance mutation; low plasmid prevalence; genomic baseline for future AMR surveillance.
Jamil et al. (2)	Adapting global guidelines to local contexts: optimizing community-acquired pneumonia (CAP) specific prescribing in Pakistan to counter antimicrobial resistance.	Qualitative interview study	National (physicians from diverse clinical settings across Pakistan).	Physicians adapt international CAP treatment guidelines based on local realities including limited diagnostic access, AMR prevalence and cost/resource constraints. Selective diagnostics and pragmatic empiric prescribing were common. Beta-lactams were prioritized for low-risk patients, macrolides and fluoroquinolones were used cautiously due to resistance concerns. Stewardship was constrained by limited diagnostics and infrastructure. Preventive strategies such as pneumococcal and influenza vaccination were underutilized due to cost and policy gaps, highlighting barriers to optimal AMR-informed CAP management.
Azra et al. (5)	Comparative genomic insights into multidrug resistance in classical and hypervirulent K. pneumoniae clinical isolates.	WGS comparative genomic analysis.	Khyber Pakhtunkhwa	Genomic diversity of K. pneumoniae with broad AMR genes (β-lactamases, PMQR, aminoglycoside, tetracycline, sulfonamide determinants), highlighting complex MDR burden.
Melnychuk et al. (14)	Notable transmitted HIV drug resistance among people who inject drugs in Pakistan.	Cross-sectional HIV DRM genotyping.	Karachi, Larkana, Peshawar, Quetta, Hyderabad	High proportion (63.8%) of transmitted HIV drug resistance among PWID; low ART testing and uptake; unsafe injections linked to DRM spread.
Torumkuney et al. (6)	Results from the Survey of Antibiotic Resistance (SOAR) 2018–21 in Pakistan: data based on CLSI, EUCAST (dose-specific) and pharmacokinetic/pharmacodynamic (PK/PD) breakpoints.	Clinical antimicrobial susceptibility surveillance study.	Shaukat Khanum Memorial Cancer Hospital and Research Centre, Lahore	Surveillance of S. pneumoniae and H. influenzae (2018–21) showed low penicillin susceptibility by low-dose criteria but high susceptibility by high-dose/IV breakpoints. Good activity for β-lactams and fluoroquinolones was observed, while macrolides, tetracyclines, and trimethoprim/sulfamethoxazole showed poor activity. Most H. influenzae isolates were β-lactamase negative and largely susceptible to key antibiotics. Continued AMR surveillance is recommended.
Saleem (10)	AMR battle in Pakistan: from national action plans to local failures.	Policy correspondence/critique	National, Pakistan	Five years after implementing the National Action Plan, Pakistan's AMR response shows major gaps. Ineffective translation of policy into practice is driven by inadequate surveillance, low healthcare professional awareness of resistance patterns, poor IPC practices, rampant antibiotic misuse and regulatory weakness. The article highlights the need for strong surveillance, awareness campaigns, enforcement of regulation, and practical stewardship efforts.
Alhusseini et al. (30)	Temporal and geographic trends in extended-spectrum cephalosporins resistance among Neisseria gonorrhoeae isolates worldwide: a systematic review and meta-analysis.	Systematic review & meta-analysis	Not specific to Pakistan (global analysis)	Pooled analysis of 252 studies from 71 countries showing overall global ESC resistance ≤ 2.5% among N. gonorrhoeae, with significant temporal and geographic variation; highlights need for continuous global surveillance but does not directly report Pakistan-specific data.
Nadeem et al. (7)	Assessment of water quality and occurrence of multidrug-resistant clinically relevant bacteria in drinking water in the twin cities of Pakistan.	Environmental microbiological surveillance study.	Drinking water from filtration plants in the Twin Cities of Pakistan (Islamabad & Rawalpindi)	Drinking water contained elevated physicochemical contaminants and coliform bacteria. A range of Gram-negative bacteria showed multidrug-resistance and extensive drug resistance, with high resistance to third-generation cephalosporins. The results demonstrate ineffective water filtration and the need for improved water quality interventions and AMR monitoring in community water sources.
Khursheed et al. (15)	Regional insights on the prevalence and antimicrobial susceptibility pattern of carbapenem and colistin-resistant gram-negative bacteria: an observational cross-sectional study from Karachi, Pakistan.	Observational cross-sectional surveillance	Indus Hospital & Health Network, Karachi, Sindh Province	Among 1,785 carbapenem-resistant gram-negative isolates collected (2022–23), 1.7 % showed colistin resistance, predominantly *Klebsiella* spp. Colistin-resistant infections occurred mainly in older male in-patients. High susceptibility was retained only for tigecycline and moderate for few other agents; many first-line antibiotics showed little to no activity. Findings indicate limited treatment options and underscore the need for strengthened AMR surveillance and stewardship in Pakistan.
Tajammul et al. (16)	Detection of *Salmonella Typhi* and blaCTX-M genes in drinking water, wastewater, and environmental biofilms in Sindh Province, Pakistan.	Environmental surveillance study	Sindh Province (Hyderabad and Karachi)	*Salmonella Typhi* DNA and blaCTX-M resistance genes were found in drinking water, wastewater, and biofilms, indicating environmental dissemination of ESBL determinants. Contaminated water may act as a reservoir and vector of resistant strains, highlighting the importance of environmental AMR surveillance for public health protection.
Maryam et al. (12)	Progress on the Global Research Agenda for Antimicrobial Resistance in Human Health in Pakistan: Findings and Implications.	Systematic-narrative hybrid literature review.	National, Pakistan	Review of 349 AMR-related studies identified concentration of research in major cities and gaps in surveillance, diagnostics, stewardship and prevention. Priority actions include strengthening AMR surveillance systems, improving diagnostic capacity, promoting judicious antibiotic use, and leveraging global partnerships to implement national AMR strategies.
Khan et al. (17)	Antimicrobial resistance profiling and molecular characterization of Salmonella enterica from retail meat and poultry products in Punjab, Pakistan.	Cross-sectional food-borne surveillance and molecular analysis.	Retail meat and poultry products, Punjab Province, Pakistan	Among 600 retail meat samples, 114 Salmonella enterica isolates (19%) were recovered, all exhibiting MDR. High resistance rates were noted for ampicillin, tetracycline, trimethoprim/sulfamethoxazole, and ciprofloxacin. Lower resistance to colistin and meropenem was observed. Resistance genes (blaTEM, tetA, sul1, qnrB) and class 1 integrons were detected, indicating significant public health risks associated with consumption of contaminated meat.
Rafique et al. (18)	Utilization of clove and cinnamon essential oils as an alternative to inhibit MDR and biofilm-producing *Escherichia coli* from raw chicken meat.	Cross-sectional microbiological and antimicrobial evaluation study.	Retail raw chicken meat, Karachi, Pakistan	Analysis of 150 chicken meat samples found 49 contaminated with *E. coli*; 90% of isolates were MDR, and 59.2% produced biofilms (31% strong producers). Clove and cinnamon essential oils inhibited 40% of strong biofilm producers. No significant correlation between resistance and biofilm formation was observed. Findings highlight the high MDR *E. coli* prevalence in poultry meat and the potential role of natural antimicrobials.
Sarwar et al. (19)	Genetic diversity and antibiotic resistance paradigm of *Enterobacterales* in animal-derived food sources: a one health disquiet.	Cross-sectional environmental & food surveillance study.	Animal-derived food supply chain (meat, dairy, poultry, fish, environmental sources) across Pakistan.	Among 905 food samples, 263 *Enterobacterales* isolates (incl. *E. coli*, K. pneumoniae, P. mirabilis, *Salmonella* spp.) were identified with variable resistance patterns to common antibiotics. Colistin and tigecycline were among the more effective agents. All isolates harbored diverse resistance and virulence genes, highlighting food-borne AMR risks and the need for One Health surveillance.
Hussain et al. (20)	Patients and Surfaces: Integrated Clinical–Environmental Surveillance of MDR Gram-Negative Bacteria in Critical-Care Units (Karachi, 2024–2025).	Cross-sectional integrated clinical and environmental surveillance.	Six ICUs and HDUs, tertiary-care hospital, Karachi, Pakistan	MDR Gram-negative species (A. baumannii 36.7%, K. pneumoniae 33.9%) were prevalent across patients and environmental surfaces. Meropenem non-susceptibility was 55% and colistin non-susceptibility was 68.6%. Carbapenemase genes (blaOXA-48-like, blaNDM) were common. Network analysis highlighted transmission hubs in ICUs/HDU settings, underscoring the need for enhanced decontamination, stewardship, and continuous molecular AMR surveillance.
Basit et al. (21)	Streptococcus pyogenes in Neonates and Postpartum Women: First Report on Prevalence, Resistance, emm Typing, and Risk Factors in Khyber Pakhtunkhwa.	Cross-sectional clinical microbiological study with molecular typing.	Tertiary care hospitals in Khyber Pakhtunkhwa Province, Pakistan	Among 384 postpartum women and neonates, 14.3% had S. pyogenes infection; isolates were highly sensitive to β-lactams but showed moderate to high resistance to certain cephalosporins, macrolides, fluoroquinolones, and tetracyclines. Diverse emm types were identified, and key risk factors included prolonged labor, premature rupture of membranes, preterm birth, and neonatal resuscitation. Findings highlight a notable burden of resistant S. pyogenes and the need for strengthened AMR surveillance and stewardship.
Ali et al. (22)	Molecular epidemiology and characterization of antibiotic resistance of Pasteurella multocida isolated from livestock population of Punjab, Pakistan.	Environmental/zoonotic microbiological surveillance study.	Livestock farms in Punjab Province, Pakistan.	Pasteurella multocida isolates from haemorrhagic septicaemia showed high resistance to trimethoprim/sulfamethoxazole and erythromycin, while enrofloxacin and ceftiofur remained relatively effective. Multiple β-lactamase resistance genes (blaTEM, blaROB-1, blaOXA-2, blaNDM) and sul2 were frequently detected. These results indicate persistent circulation of resistant P. multocida in livestock and underscore the need for ongoing AMR surveillance and rational antibiotic use in the animal sector.
Rashid et al. (23)	Investigation of bedaquiline heteroresistance among *Mycobacterium tuberculosis* isolates from Pakistan.	Phenotypic testing and whole-genome sequencing of clinical MTB isolates.	National (MDR/XDR-TB patients across Pakistan)	Bedaquiline heteroresistance was detected in 29% of *M. tuberculosis* isolates, with Rv0678 mutations being predominant. No significant links to patient demographics were found. Heteroresistance occurred even without prior bedaquiline exposure, highlighting emerging complexity in TB drug resistance and need for enhanced diagnostics and tailored treatment strategies in Pakistan.
Asghar et al. (24)	Seasonal and hospital settings variations in antimicrobial resistance among clinical isolates from cardiac patients: insights from a 7-Year study.	Retrospective cross-sectional analysis	Cardiac hospital, Faisalabad, Pakistan	Analysis of 3,035 clinical records revealed S. aureus, K. pneumoniae, and *E. coli* as dominant pathogens. Higher isolation rates were seen in inpatients with seasonal peaks. S. aureus showed complete resistance to vancomycin and oxacillin; common resistance genes (mecA, vanA, tetM, aph3′) were detected. Polymyxin B and colistin remained effective against many MDR strains. Year-round surveillance and stewardship interventions are essential to reduce AMR burden.
Ullah et al. (25)	Bacterial profiling and antibiotic resistance patterns in urinary tract infections: a microbiological analysis from Dera Ismail Khan, Pakistan.	Cross-sectional microbiological analysis	Dera Ismail Khan district, Khyber Pakhtunkhwa	Among 610 UTI samples, 42.6% were culture-positive. *E. coli* (59.6%) was the dominant pathogen. Imipenem, nitrofurantoin, and fosfomycin showed high *in vitro* activity, while all isolates were 100% resistant to ampicillin and amoxicillin–clavulanic acid. Findings highlight high AMR prevalence and the need for ongoing localized surveillance and evidence-based empirical treatment guidance.
Iqbal et al. (11)	Antibiotic utilization and resistance according to the WHO AWaRe classification in intensive care units after COVID-19 third wave in Pakistan: Findings and implications.	Retrospective observational antibiotic use and resistance analysis.	Intensive care units across hospitals in Pakistan, post third COVID-19 wave	Retrospective analysis showed high antibiotic utilization (768 prescriptions among 313 ICU patients), with predominant use of amoxicillin/clavulanic acid. Carbapenem sensitivity was higher than other classes. AWaRe classification revealed 31.8% Access, 59.5% Watch, 8.7% Reserve antibiotics. Highlights the need for stewardship to optimize antibiotic use and address AMR in ICU settings.
Ullah et al. (26)	Multiple highly resistant clones of MRSA circulating among patients with skin and soft tissue infection, Peshawar, Pakistan 2021–2022.	Observational microbiological surveillance with molecular typing.	Lady Reading Hospital, Peshawar, Khyber Pakhtunkhwa Province	A total of 154 MRSA isolates from SSTIs showed high resistance to ciprofloxacin (85.7%), erythromycin (76%), sulfamethoxazole and gentamicin (~68.8%), fusidic acid (57.8%) and tetracycline (55.8%). Molecular analysis revealed 16 MRSA lineages, with prominent CC8-MRSA-IV and PVL-positive CC1/ST772-MRSA-V clones; PVL was found in ~45.5% of isolates, indicating significant AMR and virulence concerns.
Zeb et al. (8)	Antimicrobial resistant *Brucella* spp. prevail in raw milk and animal feces of different livestock farms.	Environmental/zoonotic surveillance microbiological study.	Livestock farms in Punjab and Islamabad Capital Territory, Pakistan	*Brucella* spp. was detected in raw milk and fecal samples; isolates showed multiple antibiotic resistance with 75% classified as MDR and high MAR indices (0.3–0.5), indicating significant AMR presence in animal reservoirs and highlighting zoonotic public health risks.

### 3.3 Prevalence and trends of AMR in Pakistan

AMR in Pakistan demonstrates high prevalence and diversity across bacterial species and geographic regions. Enteric pathogens such as Salmonella Paratyphi A and Vibrio cholerae show fluoroquinolone resistance and emerging macrolide resistance (1, 4). Hospital-based surveillance of Gram-negative pathogens, including *Klebsiella pneumoniae* and Acinetobacter baumannii, highlights multidrug resistance with limited treatment options, particularly against carbapenems and colistin (5, 20).

Community-acquired pathogens such as Streptococcus pneumoniae and Haemophilus influenzae maintain high susceptibility to β-lactams under high-dose regimens but show low susceptibility to macrolides and tetracyclines (6). Environmental surveillance studies reveal contamination of water sources and livestock products with MDR and XDR strains, indicating a reservoir for resistance beyond hospital settings (7, 8).

Trends indicate sustained resistance in enteric pathogens, increasing MDR in hospital-acquired infections, and emerging resistance to key therapeutic classes. Urban tertiary care centers report higher detection rates, suggesting diagnostic bias, whereas rural data are limited. Resistance determinants are increasingly documented through whole-genome sequencing, revealing both stable lineages and ongoing mutations conferring resistance (1, 4).

Hospital-based studies consistently reported higher multidrug resistance rates than environmental surveillance studies, likely reflecting increased antibiotic exposure, critically ill patient populations, and tertiary-care sampling bias. In contrast, environmental and One Health studies demonstrated that resistant organisms and resistance genes are increasingly disseminated through water systems, livestock, and food sources. Despite methodological heterogeneity, most studies consistently identified irrational antibiotic use, limited diagnostic infrastructure, weak infection prevention practices, and fragmented surveillance systems as major contributors to the AMR burden in Pakistan.

Several studies also demonstrated substantial geographic heterogeneity in resistance patterns, with tertiary-care centers in urban provinces reporting higher prevalence of multidrug-resistant pathogens. However, data from rural regions and underrepresented provinces such as Balochistan remain limited, indicating important surveillance gaps and potential underestimation of the national AMR burden.

Recent studies have further demonstrated increasing multidrug resistance in environmental, community, and genomic surveillance settings across Pakistan (27–29).

### 3.4 Economic consequences in healthcare and productivity

The economic repercussions of AMR in Pakistan are substantial, affecting healthcare expenditures, individual patients, and national economic stability.

- Direct Healthcare Costs: Treating antibiotic-resistant blood infections incurs significant additional expenditure, estimated at approximately USD 33.97 (PKR 9483.2) per instance due to prolonged hospital stays (9). Direct costs, including laboratory tests, antibiotics, and transportation, are significantly higher for AMR patients compared to those with susceptible infections (9). No prior study had estimated the costs related to AMR in hospitals in Pakistan (9).
- Indirect Costs and Productivity Loss: Indirect costs, such as spending on food, accommodation, and productivity loss, are also higher for patients with resistant infections (9). The loss of productivity accounts for approximately one-third of the extra costs associated with AMR (9). At a macroeconomic level, AMR can lead to reduced productivity and economic output, with global projections indicating a potential annual GDP decline of 3.8% by 2050 and a shortfall of USD 3.4 trillion by 2030 (9).
- Increased Mortality: AMR is associated with increased mortality rates. One study reported 46 deaths from 12 resistant bacteria in bloodstream infections among 193 patients, with the AMR cohort experiencing the highest mortality (45.65%) (9). Resistant infections are linked to a 1.3–2 times increase in mortality, morbidity, and expense compared to susceptible infections (9).
- Economic Burden on Patients: Patients infected with resistant bacteria face a higher economic burden than those with susceptible infections, with mean total costs reaching approximately PKR 39,678 in one hospital setting (9). This includes additional costs for laboratory tests, antibiotics, and transportation (9).

Epidemiological trends in multidrug resistance among major pathogens reported in Pakistan between 2015 and 2025. The figure represents a qualitative synthesis of published surveillance and clinical studies included in this review rather than pooled national surveillance estimates. Trends were derived from reported resistance patterns in *Escherichia coli, Klebsiella pneumoniae, Salmonella Typhi*, and *Vibrio cholerae* across multiple healthcare settings.

### 3.5 Social determinants and impacts

Social factors are deeply intertwined with the prevalence and spread of AMR in Pakistan, presenting unique public health challenges.

- Inadequate Awareness and Education: A critical lack of awareness regarding AMR exists among both the general public and healthcare professionals (10). Physicians often lack knowledge of local resistance patterns, leading to treatment decisions based on outdated or generalized information (10).
- Irrational Antibiotic Use and Self-Medication: Public behaviors like self-medication with leftover antibiotics, seeking treatment from unqualified practitioners, and the easy over-the-counter availability of antibiotics significantly fuel irrational antibiotic use (10, 11, 35, 36). A cross-sectional study reported the prevalence of self-medication with antimicrobials in Pakistan at 32.5%, with ciprofloxacin being the most commonly used drug (35). This practice is driven by financial constraints, inadequate public education, and limited access to credible healthcare professionals (10, 34, 35). Incorrect antibiotic use in primary care is as high as 88% in Pakistan (9).
- Weak Regulatory Frameworks: Poor legislation, weak regulatory enforcement, and a lack of strict monitoring by the Drug Regulatory Authority of Pakistan contribute to the rampant dispensing of antibiotics without proper prescriptions (10, 11). The pharmaceutical industry's focus on branded generics and an oversaturation of the market with broad-spectrum antibiotics, often with quality concerns, exacerbate the problem (10).
- Public Trust and Healthcare Access: There is a reported decline in public trust in the health system due to AMR's impact (9). Limited access to healthcare, with only one physician per 1,300 patients, pushes many to self-medicate or delay seeking professional medical attention (35).
- Social and Behavioral Influences: High rates of illiteracy (approximately 30%) affect health literacy and the adoption of prudent antimicrobial use (35). Misleading advertisements and a high prevalence of quackery further complicate rational drug use (9).

## 4 Discussion

### 4.1 Critical interpretation of evidence

The included studies demonstrated considerable heterogeneity in study design, surveillance methodology, and sampling populations. Hospital-based surveillance may overestimate resistance prevalence due to selection of critically ill patients, whereas environmental and community-based studies may underestimate severe clinical outcomes because of limited diagnostic infrastructure and underreporting. These methodological differences should be considered when interpreting national AMR trends.

The evidence indicates that antimicrobial resistance in Pakistan is not confined to isolated outbreaks but represents a sustained and evolving public health threat. Genomic findings, such as those reported by Mylona et al. (1), provide critical baseline data demonstrating stable pathogen population structures alongside emerging resistance determinants. These findings are particularly important in the context of vaccination strategies targeting typhoid, as pathogen replacement or resistance shifts may alter disease patterns.

The persistence of fluoroquinolone resistance and the detection of azithromycin resistance markers are concerning because they compromise affordable oral treatment options. In a resource-limited health system, reliance on more expensive or intravenous therapies increases healthcare expenditure and hospital resource utilization. This dynamic creates a reinforcing cycle in which resistance increases costs, and financial constraints limit effective stewardship implementation.

**Figure 4:**
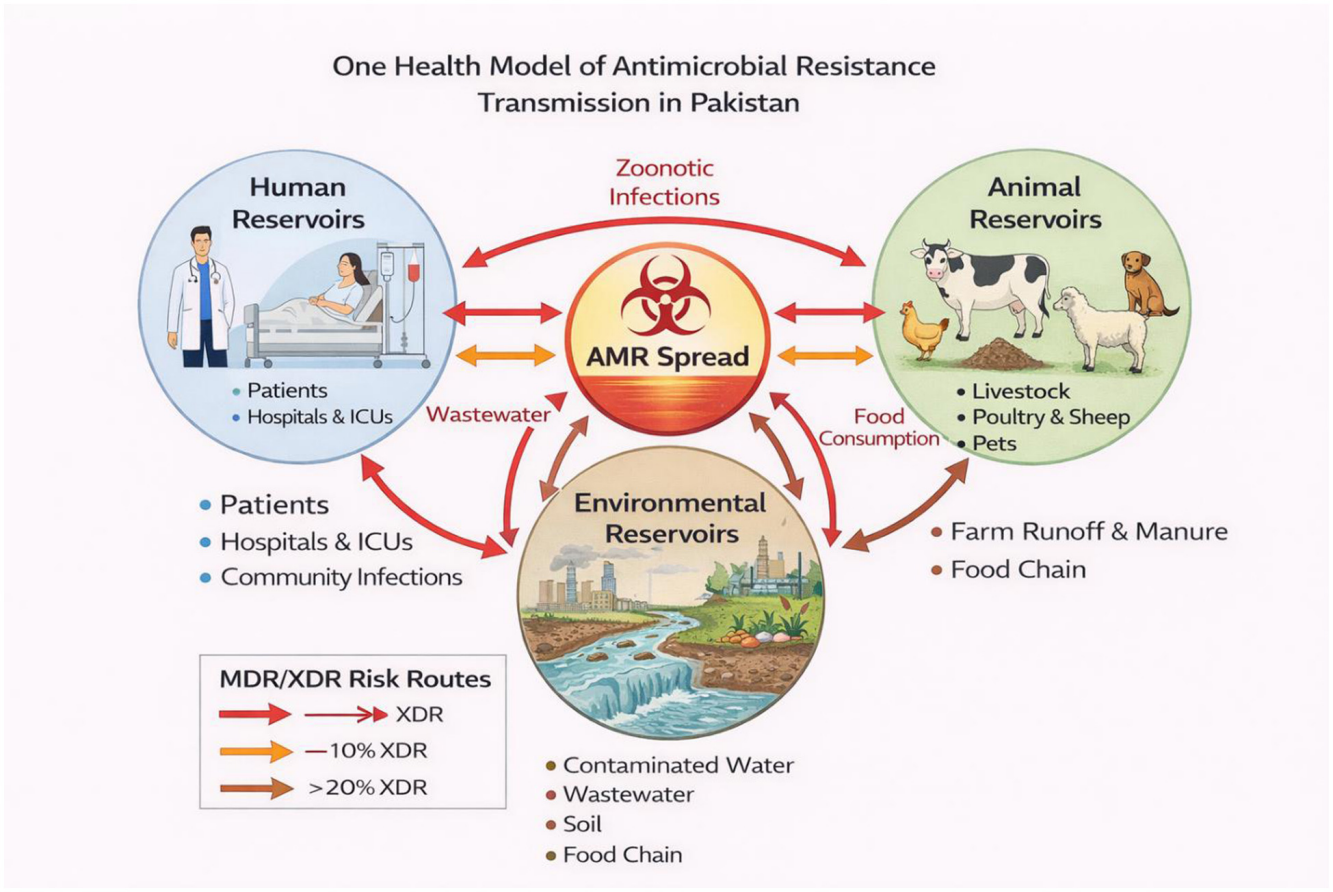
One Health conceptual framework demonstrating interactions between human, environmental, and healthcare-related contributors to antimicrobial resistance in Pakistan. This figure was developed by the author for this review.

The above Figure 4 depicts the interactions between humans, animals, and the environment in the spread of antimicrobial resistance (AMR). It highlights key reservoirs such as hospitals, communities, livestock, food, and water and illustrates bidirectional transmission pathways of resistant bacteria and genes. Drivers such as antibiotic misuse, poor infection control, and environmental contamination are included. The model emphasizes the need for integrated One Health surveillance, stewardship, and interventions to reduce AMR emergence and dissemination.

### 4.2 Existing policies and interventions

Pakistan has engaged in various initiatives to combat AMR, demonstrating commitment at national and international levels.

- National Action Plan on AMR: Pakistan launched its NAP in 2017, with the National Institute of Health as its custodian, covering human, animal, agricultural, and environmental contexts (10, 35). The second NAP emphasizes comprehensive IPC strategies, improved surveillance systems, Antimicrobial Stewardship Programs, and educational campaigns for various stakeholders (12).
- Global Collaborations: Pakistan participates in the WHO's Global Antimicrobial Resistance and Use Surveillance System and has adopted the WHO's Access, Watch, and Reserve classification to promote judicious antibiotic use (12, 35). The UN General Assembly has recommended that 70% of global antibiotic consumption should be from the Access group by 2030 (31).
- National Programs and Initiatives: Efforts include the AMR Tricycle program, point prevalence surveys on antimicrobial usage, the Pakistan Global Antibiotic Resistance Partnership, the Pakistan AMR surveillance system, and the National TB Control Program (12). Micro-level interventions involve the Antibiotic Stewardship Initiative in Pakistan and the Pakistan Antimicrobial Resistance Network (12).

### 4.3 Socioeconomic burden

Although available studies indicate that antimicrobial-resistant infections substantially increase healthcare expenditures through prolonged hospitalization, intensive-care admissions, and use of last-line antibiotics, national-level cost-of-illness data in Pakistan remain limited. The estimated increase of approximately USD 33.97 per antimicrobial-resistant bloodstream infection reported by Safdar et al. (9) was derived from a single-center cohort study and should therefore be interpreted cautiously. Larger multicenter economic evaluations are needed before extrapolating national socioeconomic burden estimates.

### 4.4 Limitations of the review

Geographical Concentration of Research: As noted in the literature, research on AMR in Pakistan tends to be concentrated in major urban centers, particularly Karachi, Lahore, and Islamabad. This means that data from rural and remote areas, including provinces like Balochistan, where AMR challenges may differ or be more severe, might be underrepresented.

Dynamic Nature of AMR: Antimicrobial resistance is a rapidly evolving phenomenon. While the documents provide recent insights (up to 2025), continuous research is necessary to capture emerging resistance patterns and new interventions.

## 5 Conclusion

Antimicrobial resistance in Pakistan represents a structurally embedded public health and economic challenge rather than an isolated clinical phenomenon. The accumulated evidence from genomic surveillance and epidemiological investigations demonstrates sustained resistance across multiple pathogens, including enteric and respiratory organisms. The persistence of fluoroquinolone resistance and emerging azithromycin resistance signals narrowing therapeutic options, raising the risk of increased treatment failure, prolonged illness, and escalating healthcare expenditure.

The socioeconomic consequences are particularly severe in Pakistan's mixed public–private healthcare system, where out-of-pocket expenditure remains substantial. Resistant infections require longer hospital stays, advanced diagnostics, and more expensive antimicrobial agents, thereby increasing direct medical costs. Indirectly, families face income loss, caregiving burdens, and long-term financial instability. At the national level, productivity losses and increased health system strain may undermine economic resilience.

While Pakistan's National Action Plan on AMR provides a structured policy response, implementation gaps limit its effectiveness. Surveillance fragmentation, inconsistent antimicrobial stewardship practices, and weak enforcement of prescription regulations constrain measurable progress. Without sustained political commitment, cross-sector coordination, and dedicated financing, resistance trends are likely to intensify.

Future efforts must prioritize integrated genomic and epidemiological surveillance, strengthened regulatory oversight of antibiotic distribution, expanded vaccination programs, and investment in public health infrastructure. Addressing AMR in Pakistan requires alignment between scientific evidence, economic policy, and health governance. Failure to act decisively risks entrenching AMR as a chronic driver of health inequity and economic vulnerability.

## 6 Policy recommendations

Strengthening national genomic surveillance systems should be prioritized to enable early detection of resistance patterns and guide evidence-based treatment guidelines. Enforcement of prescription-only antibiotic sales must be improved through regulatory reform and accountability mechanisms. Antimicrobial stewardship programs should be institutionalized across tertiary and secondary healthcare facilities, with mandatory reporting and monitoring frameworks. Investment in vaccination strategies targeting enteric and respiratory pathogens should be expanded to reduce infection incidence and antibiotic demand. Public awareness campaigns addressing inappropriate antibiotic use must be integrated into primary healthcare services. Finally, AMR should be incorporated into broader national economic planning and health financing reforms to ensure sustained funding and cross-sector coordination.
